# Genome-wide identification and characterization of microRNAs differentially expressed in fibers in a cotton phytochrome A1 RNAi line

**DOI:** 10.1371/journal.pone.0179381

**Published:** 2017-06-14

**Authors:** Qing Miao, Peng Deng, Sukumar Saha, Johnie N. Jenkins, Chuan-Yu Hsu, Ibrokhim Y. Abdurakhmonov, Zabardast T. Buriev, Alan Pepper, Din-Pow Ma

**Affiliations:** 1 Department of Biochemistry, Molecular Biology, Entomology and Plant Pathology, Mississippi State University, Mississippi State, MS, United States of America; 2 Department of Pharmacology, Weill Cornell Medical College of Cornell University, New York, NY, United States of America; 3 USDA-ARS, Crop Science Research Laboratory, Mississippi State, MS, United States of America; 4 Institute for Genomics, Biocomputing and Biotechnology, Mississippi State University, Mississippi State, MS, United States of America; 5 Center of Genomics and Bioinformatics, Academy of Sciences of Uzbekistan, Tashkent, Uzbekistan; 6 Department of Biology, Texas A & M University, College Station, TX, United States of America; Dokuz Eylul Universitesi, TURKEY

## Abstract

Cotton fiber is an important commodity throughout the world. Fiber property determines fiber quality and commercial values. Previous studies showed that silencing phytochrome A1 gene (*PHYA1*) by RNA interference in Upland cotton (*Gossypium hirsutum* L. cv. Coker 312) had generated *PHYA1* RNAi lines with simultaneous improvements in fiber quality (longer, stronger and finer fiber) and other key agronomic traits. Characterization of the altered molecular processes in these RNAi genotypes and its wild-type controls is a great interest to better understand the *PHYA1* RNAi phenotypes. In this study, a total of 77 conserved miRNAs belonging to 61 families were examined in a *PHYA1* RNAi line and its parental Coker 312 genotype by using multiplex sequencing. Of these miRNAs, seven (miR7503, miR7514, miR399c, miR399d, miR160, miR169b, and miR2950) were found to be differentially expressed in *PHYA1* RNAi cotton. The target genes of these differentially expressed miRNAs were involved in the metabolism and signaling pathways of phytohormones, which included Gibberellin, Auxin and Abscisic Acid. The expression of several MYB transcription factors was also affected by miRNAs in RNAi cotton. In addition, 35 novel miRNAs (novel miR1-novel miR35) were identified in fibers for the first time in this study. Target genes of vast majority of these novel miRNAs were also predicted. Of these, nine novel miRNAs (novel-miR1, 2, 16, 19, 26, 27, 28, 31 and 32) were targeted to cytochrome P450-like TATA box binding protein (TBP). The qRT-PCR confirmed expression levels of several differentially regulated miRNAs. Expression patterns of four miRNAs-targets pairs were also examined via RNA deep sequencing. Together, the results imply that the regulation of miRNA expression might confer to the phenotype of the *PHYA1* RNAi line(s) with improved fiber quality.

## Introduction

Cotton (*Gossypium hirsutum* L.) is one of the most important cash crops around the world, providing the largest renewable natural fiber for the textile industry. The cotton fiber is a trichome cell derived from the epidermal layer of the ovule, and its development consists of four overlapping stages: initiation, elongation (primary wall biosynthesis), secondary wall biosynthesis and maturation [1]. Previous studies showed that silencing phytochrome A1 gene (*PHYA1*) by RNA interference generated RNAi cotton lines with improved fiber quality (longer, stronger and finer fiber) and vigorous shoot and root development, without adverse effects on the key agronomic traits (maturity and productivity) when compared to non-transformed Coker 312 [2]. In addition, field-trials of RNAi genotypes revealed increased photosynthesis and better adaptations to abiotic environmental stresses of RNAi genotypes compared to wild-types, a finding that may be associated with improved root development in *PHYA1* RNAi cotton plants [3].

Phytochromes are red and far-red light photoreceptors, and a high ratio of far-red light to red light perceived by the photoreceptors increased fiber length [4]. Previous molecular mapping using phytochrome-based cleaved amplified polymorphisms (CAPS and dCAPs) markers suggested an importance of cotton phytochromes on regulation of some fiber quality traits including fiber length [2, 5, 6]. Phytochromes were reported to mediate plant hormone signaling through direct interaction between their negative regulators phytochrome-interacting factors (PIFs) and certain phytohormones signaling components, such as DELLA proteins [7]. It was well known that phytohormones play central roles in the regulation of cotton fiber development. Auxin, gibberellins (GAs), bassinosteroids (BR), and ethylene (ET) have been shown to promote fiber cell initiation and fiber development [8–11], whereas abscisic acid (ABA) and cytokines (CK) negatively affect fiber development [12]. Over the past decades, many genes have been reported to be involved in cotton fiber development. Genes encoding MYB transcription factors and sucrose synthase play important roles in fiber cell fate determination and fiber development [13]. Genes for cellulose synthases [14], vacuolar invertase, aquaporin, and lipid transfer proteins also play critical roles in the process of rapid fiber elongation [13] [15–17]. Calcium and the second messenger molecule H_2_O_2_ might function as the terminal signal to the fiber elongation [18].

MicroRNAs are a family of small (21–25 nt) noncoding RNA molecules that regulate eukaryotic gene expression post-transcriptionally in a sequence-specific manner. The miRNA gene (MIR) is first transcribed into a larger primary transcript (pri-miRNA) by RNA polymerase II (pol II) [19]. The pri-miRNA is then processed into a 60–120 nt pre-miRNA with a stem-loop structure [20]. The pre-miRNA is further processed to form a miRNA:miRNA* duplex. In plants, these two successive cleavages occur in the nucleus and are facilitated by the dicer enzyme. The miRNA:miRNA* duplex is exported into cytoplasm by HASTY [21]. The RNA strand with the weakest 5´ end base pairing in the duplex is then selected as the mature miRNA, and the other strand, called miRNA* is degraded [22]. The mature miRNA is loaded to RISC (RNA-induced silencing complex) and guided by ARGONAUTE proteins (as parts of RISC) to target mRNA complementary sequences and trigger mRNA cleavage or translational inhibition [23]. miRNAs play regulatory roles in many aspects of plant development, hormone signaling, abiotic stress resistance such as heat, salinity and drought [24, 25]. More interestingly, miRNAs have also been shown to play crucial roles in cotton fiber development [26].

MicroRNA identification in cotton is lagging behind other plant species such as *Arabidopsis* and rice, which might be due to polyploidization events occurred in the cotton genome. *Gossypium hirsutum*, also known as upland cotton, is an allotetraploid (AADD, 2n = 4x = 52), arose from hybridization between a transoceanic dispersal of an A-genome progenitor (*Gossypium arboretum*, AA, 2n = 2x = 26) and a local D-genome progenitor (*Gossypium raimondii*, DD, 2n = 2x = 26). The genome sequences of two diploid cotton *Gossypium raimondii* [27] and *Gossypium arboretum* [28], and the allotetraploid *Gossypium hirsutum* [29, 30] have been determined. Whole genome sequences will definitely accelerate the identification and annotation of cotton miRNAs, especially cotton-specific miRNAs. Cotton miRNAs were first identified in 2007 by two groups using a comparative genomic approach [31, 32]. Thirty and thirty-seven miRNAs candidates were identified by the two groups, respectively. Besides homology searches on database, Abdurakhmonov et al. [33] used a direct cloning technique to identify three miRNAs in cotton. Recently high-throughput RNA sequencing have been adopted for identification and expression analysis of miRNAs in cotton. Through small RNA sequencing, 34 conserved miRNAs families were identified in *G*. *hirsutum* [34] and 22 conserved miRNA families were expressed in developing cotton ovules [35]. By using the same approach, Pang et al. identified 4 novel miRNA families and proved that miRNAs play a role in cotton fiber development [26]. Recently, 65 conserved miRNA families were identified in cotton leaf and ovule tissues and forty of the identified miRNAs were ovule specific, suggesting that these miRNAs may play important roles in ovule and fiber development [34, 36]. Using short fiber mutants, Naoumkina et al. [37] identified 24 conserved and 147 novel miRNA families and revealed that 4 miRNAs were involved in fiber elongation. In comparison to other plant species such as *Arabidopsis* or rice, less miRNAs had been identified in cotton. With its larger genome size, it is predicted that the cotton genome may encode more than the typical number of miRNAs, including some with unique roles in fiber development.

With the aim to better understand the altered molecular pathways in the *PHYA1* RNAi line of tetraploid cotton, and because of potential importance of miRNAs in the complex developmental processes such as fiber quality, miRNA libraries were constructed in this study using fiber RNAs (at different development stages) extracted from the *PHYA1* RNAi cotton line and its non-transgenic background Coker 312. The libraries were subjected to multiplex sequencing on the Illumina platform, and conserved and novel miRNAs were then identified and used for differential expression analysis. As results, a total of 77 known miRNAs from 61 different families and 35 novel miRNAs were identified in developing cotton fibers. Of these, 7 miRNAs were differentially expressed in the *PHYA1* RNAi line. Our results implied that the regulation of miRNA expression might play an important role in cotton fiber development.

## Materials and methods

### Plant materials and plant growth

*Gossypium hirsutum* L. cv. Coker 312 and its phytochrome A1 (*PHYA1*) RNAi plants were grown in the field at the North Farm R. R. Foil Plant Science Research Center of Mississippi State University. The seeds of these RNAi cotton plants [2] were provided by Uzbekistan collaborator of this study through Technology Transfer Office of USDA-ARS, USA under the USDA-Uzbekistan cooperation programs. Flowers were tagged on the day of anthesis (0 DPA). Cotton bolls (5, 10, 15 DPA) were harvested with three biological replications. Fibers were carefully dissected from cotton bolls, immediately frozen in liquid nitrogen, and then stored at -80°C for small RNA extraction.

### Isolation of small RNAs and library construction

The small RNAs were isolated from fiber tissues by using the mirPremier™ MicroRNA Isolation Kit (Sigma-Aldrich) according to the manufacturer’s instructions. The RNA concentration and purity were determined by using a Nanodrop 2000c spectrophotometer (ThermoFisher Scientific). The Next Multiplex Small RNA Library Prep Set (New England Biolabs) was used to convert isolated RNAs (500 ng) into barcoded small RNA libraries according to the manufacturer’s instructions. To determine the differential expression of miRNAs in fibers between the PHYA1 RNAi line and its non-transgenic background line Coker 312, 18 barcoded small RNA libraries with three biological replicates were constructed by using miRNAs extracted from fibers of the two lines at three different developmental stages of 5-, 10-, and 15-DPA, respectively. Size selection (~150 nt) of the libraries was performed using AMPure XP beads. The concentration and quality of the libraries were determined with a Qubit Fluorimeter (ThermoFisher Scientific) and the Bioanalyzer 2100 (Agilent Technologies). The libraries were pooled and single-end (1 X 50) sequenced on an Illumina HiSeq 2500 system performed by BGI. The sequencing reads were deposited into NCBI Sequence Read Archive (SRA) with accession number SRX2467209-17 (*Gossypium hirsutum* L. cv. Coker 312) and SRX2467222-30 (RNAi line), respectively. Data files are available at https://www.ncbi.nlm.nih.gov/Traces/study/?acc=SRP096134 (Coker 312) and https://www.ncbi.nlm.nih.gov/Traces/study/?acc=SRP096136 (RNAi line).

### MicroRNA identification and target prediction

Total raw sequences generated from Illumina instrument were trimmed to remove low-quality reads and adaptor sequences using Trimmomatic [38], and FastQC (http://www.bioinformatics.bbsrc.ac.uk/projects/fastqc/) was used to test data quality. The miRBase database release 21 (http://www.mirbase.org/) and the *Gossypium hirsutum* L. acc. TM-1 genome sequence [30] were used as references for known conserved miRNA mapping and novel miRNA identification, respectively. SeqMan NGen and ArrayStar (DNASTAR, Inc.) were used for mapping of clean short reads (18–44 nt) to the reference and expression level analysis. The miRNA expression was normalized by the Reads Per Kilobase per Million mapped reads (RPKM) method [39].

Conserved miRNAs were identified by comparing to miRBase with the criterion that tags were similar to their homologues within two mismatches and without gaps [40]. Potential novel miRNAs were firstly BLAST verified against non-coding rRNA, tRNA, small nuclear RNA (snRNA), and small nucleolar RNA (snoRNA) in GenBank, tRNA database (http://gtrnadb.ucsc.edu), and Rfam database [41]. Sequences with no hits to known miRNAs or other cellular RNAs were mapped to the *Gossypium hirsutum* L. acc. TM-1 genome. The mireap 0.2 (https://sourceforge.net/projects/mireap/) was used to analyze pre-miRNAs [42]. The psRNATarget was used for target prediction [43]. Sequence folding prediction were made by MFOLD [44]. miRNAs with negative hairpins folding energy from -25.7 to -230.5 kcal mol^-1^ [45] were selected for further analysis.

### Quantitative real-time PCR

Quantitative Real time PCR was performed to validate the expression of miRNAs identified from RNA seq. The isolated fiber small RNAs (500 ng) were first reverse-transcribed to generate cDNAs using a Mir-X miRNA First-Strand Synthesis Kit (Clontech Laboratories). The cDNAs were then used as template for quantitative real-time PCR on LightCycler 480 (Roche Holding AG) by using SYBR Advantage qPCR Premix (Clontech Laboratories). The qRT-PCR was carried out by pre-denaturation at 95°C for 15 seconds, following by 45 cycles of 3-step PCR with denaturation at 95°C for 5 seconds, annealing at 55–60°C for 15 seconds, and final extension at 72°C for 15 seconds. Gene expression levels were presented as fold-change and calculated using the comparative threshold cycle (C_T_) method as described [46] with U6 snRNA as the internal reference. The expression data of four target genes (Gh_Sca142710G01, Gh_Sca006071G01, Gh_A05G2828 and GhD07G0477) were extracted from high throughput RNA-seq for 10 DPA fibers of RNAi line and Coker 312 (unpublished data). All the primers used for miRNA qRT-PCR were listed in Supplementary S1 Table.

## Results

### High-throughput deep sequencing of 5-, 10- and 15-DPA fiber small RNA libraries from both Coker 312 and *PHYA1* RNAi plants

The libraries were subject to next generation sequencing on HiSeq 2500. Libraries had a minimum 8,052,792 and a maximum 12,947,430 short reads extracted from raw data (Table 1). Adaptor sequences and low-quality reads composed of 39.54% ~ 65.63% of raw reads were removed, and the average length of short reads after trim was from 22.5 to 24.2 nt (Table 1). Within the trimmed reads, 8.9%, 8.9%, 9.6%, 10.3%, 10.7% and 10.4% were annotated for 5 DPA (CF5), 10 DPA (CF10) and 15 DPA (CF15) fiber from Coker 312 and 5 DPA (RF5), 10 DPA (RF10) and 15 DPA (RF15) fiber from the *PHYA1* RNAi line, respectively (Table 2). Among all the reads, 6,566,588; 6,588,884; 5,025,362; 8,286,014; 6,426,397; and 4,741,820 unique reads were annotated as small RNAs, respectively (Table 2). Most of these reads were clustered into rRNA, tRNA, snRNA, snoRNA and scRNA and removed before miRNA analyses. Among the rest of the annotated reads, 7,004, 7,291, 5,704, 8,698, 6,942, and 5,559 reads were matched to miRBase database (Table 2). The size distribution of these small RNAs ranged from 15 to 30 nt, and the most abundant length of them was 24 nt in both Coker 312 and RNAi lines (S1 Fig).

**Table 1:** Trim reads statistics from raw data image file.

Sample ID	Raw reads	Trimmed reads	Trimmed percentage (%)	Average length after trim
CF5-1	8,052,792	3,867,782	48.03	22.8
CF5-2	11,192,002	7,345,455	65.63	22.7
CF5-3	10,445,749	6,503,668	62.26	22.6
CF10-1	10,496,469	6,475,658	61.69	22.9
CF10-2	10,807,969	7,164,085	46.45	22.6
CF10-3	10,343,483	4,804,740	46.45	22.7
CF15-1	9,721,703	4,083,368	42	22.5
CF15-2	11,898,328	5,846,551	49.14	22.7
CF15-3	12,947,430	6,272,361	48.44	23.0
RF5-1	8,684,895	5,396,278	62.13	23.3
RF5-2	12,917,341	7,671,999	59.39	23.4
RF5-3	12,783,126	8,366,374	65.45	23.3
RF10-1	10,924,895	5,315,759	48.66	23.7
RF10-2	10,578,419	5,777,184	62.69	24.2
RF10-3	10,347,993	6,487,061	62.69	24.2
RF15-1	10,939,628	5,511,637	50.38	23.4
RF15-2	11,395,084	5,859,236	51.42	23.2
RF15-3	10,194,345	4,040,911	39.54	23.7

Note: RF and CF denote fibers from RNAi and Coker 312 lines, respectively. The numbers 5, 10, 15 represent the days after anthesis. Each of CF and RF samples had 3 biological replicates (1–3).

**Table 2:** Distribution of mapped sequence reads.

Category	CF5	CF10	CF15	RF5	RF10	RF15
Total reads	17,716,905	18,444,483	16,202,280	21,434,651	17,580,004	15,411,784
Annotated	1,583,027(8.9%)	1,645,910(8.9%)	1,554,366(9.6%)	2,214,010(10.3%)	1,883,895(10.7%)	1,607,358(10.4%)
Total Identified Small RNAs	6,566,588	6,588,884	5,025,362	8,286,014	6,426,397	4,741,820
Annotated Small RNAs	373,566(5.7%)	354,203(5.4%)	308,890(6.1%)	506,395(6.1%)	390,173(6.1%)	298,707(6.3%)
Small RNAs Match miRBase	7,004	7,291	5,704	8,698	6,942	5,559
Small RNAs Match *Gossypium hirsutum*	366,562	346,912	303,186	497,697	383,224	293,148

Note: RF and CF denote fibers from RNAi and Coker 312 lines, respectively. The numbers 5, 10, 15 represent the days post anthesis.

### Identification of differentially expressed known miRNAs in *PHYA1* RNAi cotton fibers

**Fig 1:**
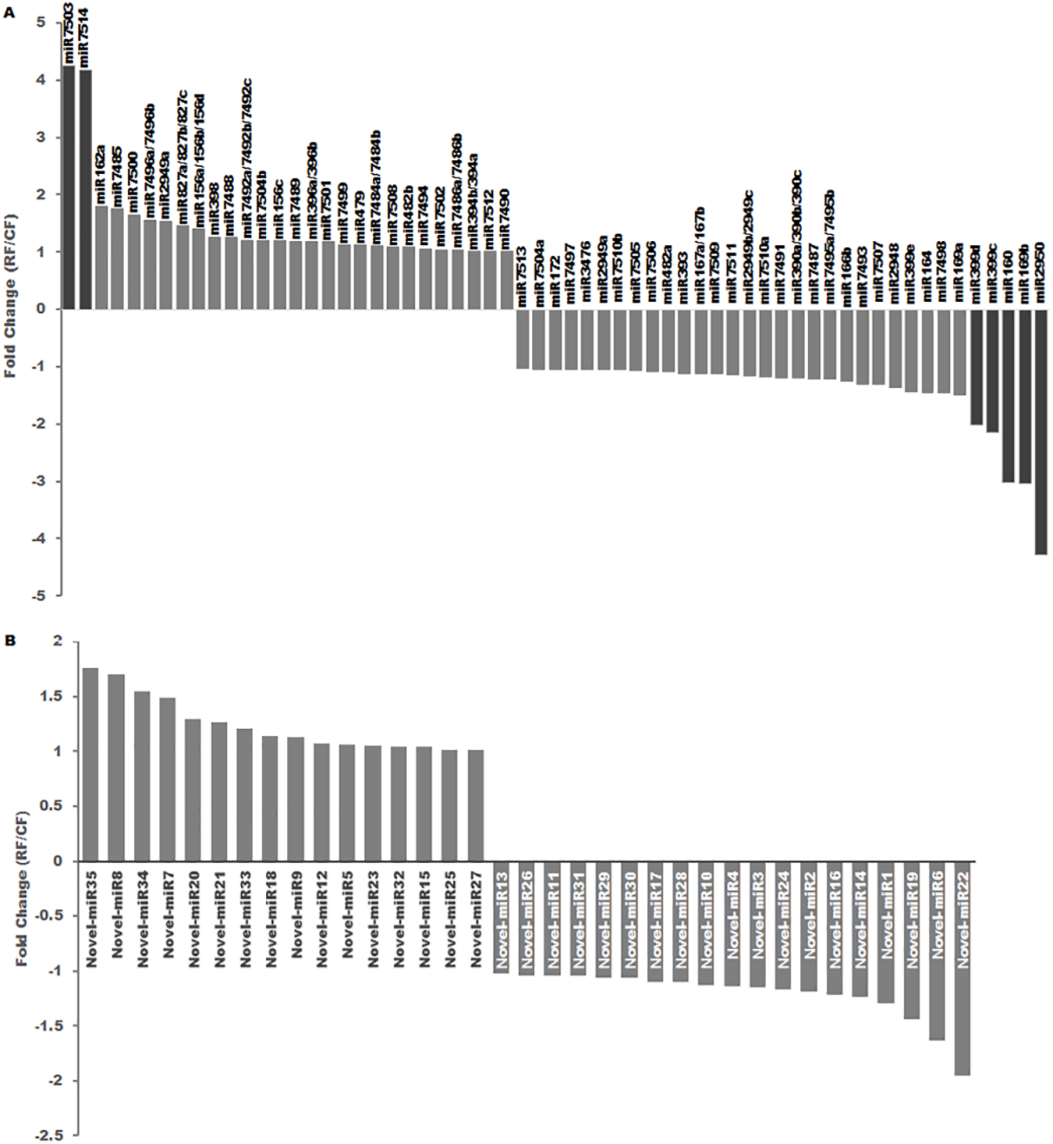
**Differentially-expressed-known miRNA families (A) and novel miRNAs (B) in *PHYA1*-RNAi cotton (RF) relative to the non-transformed Coker 312 (CF) in fibers.** Those with a fold change greater than 2 are displayed by darker columns.

Known and conserved miRNAs were identified by mapping to the miRBase database (release 21), and 77 known miRNAs belonging to 61 miRNA families were found and they are all present in both of the two cotton lines. Of these, 34 miRNA families were cotton-specific, and 7 miRNAs were differentially expressed (≥ 2-fold change, FDR ≤ 0.05) in the *PHYA1* RNAi cotton. For majority of the miRNA families, only one member was identified. However, multiple members were identified in some miRNA families, which included miR7495, 390, 2949, 167, 399, 7486, 394, 7484, 396, 7492, 156, 827, and 7496 (Fig 1A).

Among these conserved miRNAs, 39 miRNAs were up-regulated in *PHYA1* RNAi compared to Coker 312, and 38 miRNAs were down-regulated in the RNAi line (Fig 1A). Seven of conserved miRNAs, including miR7503, miR7514, miR399c, miR399d, miR160, miR169b and miR2950, were differentially expressed (fold change ≥ 2) in the *PHYA1* RNAi line compared to Coker 312. It will be interesting to determine whether miRNA-mediated gene regulation could confer to the phenotype of the RNAi line with improved fiber quality.

### Identification of novel miRNAs in *PHYA1* RNAi cotton fibers

In addition to successful identification of 77 conserved miRNAs in the *PHYA1* RNAi and wild type lines, novel miRNAs in fibers were also identified in both RNAi and wild-type cotton lines. After removing known miRNAs and other small RNAs, the stem-loop secondary structure of novel pre-miRNAs was predicted by MFOLD. The stability of an RNA secondary structure is affected by the minimal folding free energy (MFE). The lower the MFE value, the more stable the RNA structure. One of the criteria to annotate a novel miRNA is that it was generated by precise excision from the stem of a stem-loop precursor [47]. A total of 35 novel miRNAs (named as novel-miR 1 to 35) were identified in the 18 fiber libraries (Table 3). The sizes of these novel miRNAs ranged from 21 to 24 nt. The 24 nt miRNAs were the most abundant with 27 newly discovered miRNAs. Five, two and one novel miRNAs were found to have the sizes of 21, 22 and 23 nt, respectively. The stem-loop precursors of these novel miRNAs predicted by MFOLD have negative free folding energies from -45.7 to -230.1 kcalmol^-1^, which are lower than those of tRNAs (-27.5 kcal mol^-1^) or rRNA (-33 kcal mol^-1^) (Table 3). All miRNAs* of these novel miRNAs have been identified in RNA sequencing. Among these novel miRNAs, none were significantly differentially expressed in *PHYA1* RNAi fibers (≤ 2 fold change) (Fig 1B). The 35 novel miRNAs were identified in cotton fibers for the first time, and their mature miRNA sequences were listed in Table 3.

**Table 3:** Novel miRNAs identified in all libraries.

Name	Sequence	Length	MFE	Accession number	Annotation	Inhibition	Cleavage/Translation inhibition
		(nt)	(kcal/mol)	(NBI)			site (nt)
Novel-miR1	CCGGAGACGTCGGCGGGGGCCTCG	24	-215.5	Gh_Sca142710G01	RRNA promoter binding protein	Cleavage	497
				Gh_D08G0862	Cytochrome P450 like_TBP	Cleavage	945
				Gh_Sca014836G01	atp synthase subunit beta	Cleavage	408
				Gh_D11G1394	chaperonin cpn60- mitochondrial	Cleavage	993
				Gh_A05G2828	senescence-associated protein	Cleavage	293
Novel-miR2	CCGACTGTTTAATTAAAACAAAGT	24	-158.2	Gh_D08G0862	Cytochrome P450 like_TBP	Cleavage	916
				Gh_Sca009741G01	Cytochrome P450 like_TBP	Cleavage	1223
				Gh_D08G0858	Cytochrome P450 like_TBP	Cleavage	530
				Gh_Sca006071G01	elongation factor 1-alpha	Cleavage	1198
Novel-miR3	CGGCAAAATAGCTCGACGCCAGGA	24	-72.3	Gh_D04G2001	ORF107c	Cleavage	429
Novel-miR4	GGCTCAGCCGGAGGTAGGGTCCAG	24	-230.1	Gh_A05G2828	senescence-associated protein	Cleavage	214
Novel-miR5	CCGACCTTAGCTCAGTTGGTAGA	23	-218.3	Gh_A02G0253	PE-PGRS FAMILY PROTEIN	Cleavage	240
Novel-miR6	AAGAATTTGGGCTTTTGTGACTCG	24	-190.7	Gh_D04G0695	N/A	Cleavage	362
				Gh_A01G0034	N/A	Cleavage	665
Novel-miR7	CCAAGATCAATAGACAGGCGTG	22	-203.2	Gh_D06G0864	alpha-aminoadipic semialdehyde synthase	Cleavage	308
				Gh_D12G1259	BZIP transcription factor-like protein	Translation	146
Novel-miR8	TTCCACAGCTTTCTTGAACTT	21	-95.1	Gh_D07G0477	chaperone protein chloroplastic	Cleavage	18
				Gh_D02G2222	calcium-binding ef-hand family protein	Cleavage	346
				Gh_D08G0162	serine threonine-protein kinase nek6	Cleavage	582
				Gh_D05G3256	bacterial-induced peroxidase precursor	Cleavage	1166
Novel-miR9	TGGATGGGCGATTGGGTGCGGTGC	24	-192.5	Gh_D07G1749	14-3-3-like protein	Cleavage	230
Novel-miR10	ATTATCCGATGAGGCACTGGGTGC	24	-141.3	Gh_A08G2090	eukaryotic translation initiation factor isoform 4g-1-like isoform x1	Cleavage	677
				Gh_A10G1238	acetolactate synthase chloroplastic-like	Cleavage	591
				Gh_D11G2202	TINY-like protein	Cleavage	493
Novel-miR11	ATTTGTGGATCTATTTGACAGT	22	-174.9	Gh_A04G0528	gdsl esterase lipase at5g03610-like	Cleavage	353
				Gh_A04G0245	cbs domain-containing protein cbsx5-like	Cleavage	823
				Gh_Sca027989G01	Chloroplast 30S ribosomal protein S16	Cleavage	356
				Gh_A06G2111	endo- -beta-glucanase	Cleavage	903
				Gh_D08G2188	gtp-binding protein sar1a	Translation	1076
				Gh_D01G0099	global transcription factor group isoform 1	Translation	142
Novel-miR12	GTTCGGGAGTCGGTTACGCGCGAG	24	-203.3	Gh_A02G0261	Oleosin isoform	Cleavage	501
				Gh_D11G3242	Emb|CAB10291.1	Cleavage	124
Novel-miR13	TTTGTACTTTAGATGTCTCTC	21	-62.3	Gh_D11G1863	Membrane protein-like protein	Cleavage	235
				Gh_D12G1850	Receptor-like protein kinase	Translation	239
				Gh_D02G1964	3-hydroxy-3-methylglutaryl-coenzyme A reductase	Cleavage	294
				Gh_A05G3942	40s ribosomal protein s3-3	Cleavage	37
				Gh_D10G2028	Mitochondrial carrier protein, expressed	Cleavage	454
Novel-miR14	GACGGACTGGGAACGGCTCCC	21	-164.3	N/A	N/A	N/A	N/A
Novel-miR15	AATCGGTTGGCATGTGGCACAATT	24	-224	Gh_D05G0643	probable xyloglucan glycosyltransferase 5	Cleavage	1336
Novel-miR16	AAGGATTGGCTCTGAGGGCTGGGT	24	-157.7	Gh_A05G2834	Cytochrome P450 like_TBP	Cleavage	893
				Gh_D08G0862	Cytochrome P450 like_TBP	Cleavage	661
Novel-miR17	GCGGACCAAGATCGAGTTGCCAAG	24	-220.5	Gh_D11G0625	N/A	Cleavage	107
				Gh_D05G2413	FRO1 and FRO2-like protein	Translation	227
Novel-miR18	TGAAGCTGCCAGCATGATCTC	21	-68.8	Gh_A13G0162	lim domain-containing protein wlim2b	Cleavage	513
				Gh_A06G0984	tubulin alpha-3 chain	Cleavage	89
				Gh_A02G1655	protein mid1-complementing activity 1 isoform x1	Translation	124
Novel-miR19	TCTGAGGGCTGGGCACGGGGGTCT	24	-148	Gh_A05G2834	Cytochrome P450 like_TBP	Cleavage	883
				Gh_D08G0862	Cytochrome P450 like_TBP	Cleavage	651
Novel-miR20	GGATTGTAGTTCAATTGGTCAGAG	24	-86.5	Gh_A09G1967	Bet1-like SNARE 1–2	Cleavage	177
					Predicted protein	Cleavage	610
Novel-miR21	ATTTGACGGTACCAATAATTTTGG	24	-219.2	Gh_A10G1317	N/A	Cleavage	374
				Gh_A06G2074	Octicosapeptide/Phox/Bem1p	Cleavage	769
Novel-miR22	TGTGTCAAATCGGCGGCTACATCT	24	-76.3	N/A	N/A	N/A	N/A
Novel-miR23	AATCGTCTCAATCGGACAACCGAG	24	-115.2	Gh_D03G1527	N/A	Cleavage	1938
Novel-miR24	CGGCTGCCGGCGCACTGCTCGAGT	24	-146.4	N/A	N/A	N/A	N/A
Novel-miR25	CCCAGTCCCGAACCCGTCGGCTTT	24	-149.6	Gh_A03G1313	N/A	Cleavage	738
				Gh_A05G0906	receptor-like serine threonine-protein kinase ncrk isoform x3	Cleavage	457
Novel-miR26	TAGTCCGACTTTGTGAAATGACTT	24	-45.7	Gh_Sca009741G01	Cytochrome P450 like_TBP	Cleavage	343
				Gh_D08G0862	Cytochrome P450 like_TBP	Cleavage	346
				Gh_Sca006071G01	elongation factor 1-alpha	Cleavage	526
				Gh_A05G4003	Cytochrome P450 like_TBP	Cleavage	1026
Novel-miR27	TAAACGGCGGGAGTAACTATGACT	24	-107.1	Gh_Sca009741G01	Cytochrome P450 like_TBP	Cleavage	525
				Gh_D08G0862	Cytochrome P450 like_TBP	Cleavage	528
				Gh_A05G2834	Cytochrome P450 like_TBP	Cleavage	588
				Gh_D08G0858	Cytochrome P450 like_TBP	Cleavage	736
				Gh_Sca006071G01	elongation factor 1-alpha	Cleavage	1086
				Gh_A05G4003	Cytochrome P450 like_TBP	Cleavage	1208
Novel-miR28	CAGGTCTCCAAGGTGAACAGCCTC	24	-151.1	Gh_Sca142710G01	RRNA promoter binding protein	Cleavage	296
				Gh_A05G2834	Cytochrome P450 like_TBP	Cleavage	976
				Gh_D08G0862	Cytochrome P450 like_TBP	Cleavage	744
				Gh_Sca014836G01	atp synthase subunit beta	Cleavage	207
				Gh_D08G0862	Cytochrome P450 like_TBP	Cleavage	1193
Novel-miR29	GTTGGTCGATTAAGACAGCAGGAC	24	-89.8	Gh_Sca014836G01	atp synthase subunit beta	Cleavage	287
				Gh_D11G1394	chaperonin cpn60- mitochondrial	Cleavage	1426
Novel-miR30	CACGGAATCGAGAGCTCCAAGTGG	24	-93.1	Gh_Sca014836G01	atp synthase subunit beta	Cleavage	638
				Gh_D11G1394	chaperonin cpn60- mitochondrial	Cleavage	1543
Novel-miR31	ATGTAGGCAAGGGAAGTCGGC	21	-156.3	Gh_A05G2828	senescence-associated protein	Cleavage	23
				Gh_A05G2834	Cytochrome P450 like_TBP	Cleavage	941
				Gh_D08G0862	Cytochrome P450 like_TBP	Cleavage	709
				Gh_Sca014836G01	atp synthase subunit beta	Cleavage	172
Novel-miR32	AACAGTCGACTCAGAACTGGTACG	24	-151.4	Gh_D08G0862	Cytochrome P450 like_TBP	Cleavage	676
				Gh_A05G2834	Cytochrome P450 like_TBP	Cleavage	735
Novel-miR33	ATTTCCGTAAGACATTTTCCGTGC	24	-133.4	Gh_D11G2331	Os01g0692600 protein	Cleavage	1344
				Gh_A08G1457	boi-related e3 ubiquitin-protein ligase 1-like	Cleavage	375
				Gh_D07G1808	Zinc finger, N-recognin; WD40-like	Cleavage	148
				Gh_A01G2124	60s ribosomal protein l17-2	Cleavage	162
				Gh_A07G1464	protein sulfur deficiency-induced 1-like	Cleavage	636
				Gh_A01G0147	random slug protein 5	Translation	549
				Gh_D06G1877	serine arginine repetitive matrix protein 2	Cleavage	880
				Gh_A03G1542	2-oxoglutarate/malate translocator	Translation	1181
Novel-miR34	GTGTGACTCAAATTCTAAGAGATT	24	-188.1	Gh_D02G0923	Os09g0462400 protein	Translation	181
				Gh_D02G1201	Far-red impaired response protein	Cleavage	486
				Gh_D08G0976	Arsenical pump membrane protein	Cleavage	345
				Gh_D02G0487	Protein FBL4	Cleavage	29
Novel-miR35	AGTGGAACCTCTCATTTAAGACGT	24	-210.5	Gh_A09G2497	Gag-pol	Cleavage	42
				Gh_A01G1980	At3g51780/ORF3	Translation	651
				Gh_D10G0595	Urophorphyrin III methylase	Cleavage	9

### Prediction and annotation of targets for novel miRNAs

A plant miRNA generally has perfect or near perfect complementarity to its target, and the cleavage happens between the 10^th^ and 11^th^ nucleotides from the 5´ end of the miRNA. These characters were used to identify the targets of novel miRNAs [48]. By using psRNATarget, a total of 97 targets were predicted for the 35 novel miRNAs (Table 3). Among them, the targets of three novel miRNAs novel-miR14, 22, and 24 were not identified. The top-rated target genes for each of the novel miRNAs were listed in Table 3. The details of the miRNA silencing its target and the predicted cleavage/translation inhibition sites were also presented in Table 3. Among these targets, the gene encoding cytochrome P450-like TATA box binding protein (cytochrome P450-like TBP) has the highest hits, being the target of 9 different novel miRNAs which included novel-miR1, 2, 16, 19, 26, 27, 28, 31 and 32. Plant cytochrome P450s are involved in a wide range of biosynthetic reactions, including fatty acid conjugation, hormones synthesis, and generating defensive compounds. Of these 9 novel miRNAs, only novel-miR27 and novel-miR32 were accumulated slightly higher in the *PHYA1*RNAi line, and the other 7 miRNAs, novel-miR1, 2, 16, 19, 26, 28, and 31, were all slightly lower in RNAi cotton compared to Coker 312. The novel miRNAs were also predicted to silence an rRNA promoter binding protein and some transcription factors and related proteins, such as a bZIP transcription factor, a global transcription factor group isoform 1 and a WD40-like transcription factor. Genes for ribosomal proteins were also targeted by these novel miRNAs. In addition, many proteins related to translation, such as translation initiation factor and elongation factor 1α were predicted to be the targets of novel-miR2, 10, 26 and 27. The predicted targets of these novel miRNAs also include many enzymes such as ATP synthase, serine threonine-protein kinase, peroxidase, acetolactate synthase, lipase, endo-β-glucanase, E3 ubiquitin protein ligase, receptor-like protein kinase, CoA reductase, xyloglucan glycosyltransferase, methylase, and some functional proteins, like chaperons, senescence-associated proteins, calcium-binding proteins and the 14-3-3-like protein (Table 3).

### Validation of differentially expressed miRNAs in *PHYA1* RNAi line by qRT-PCR

**Fig 2:**
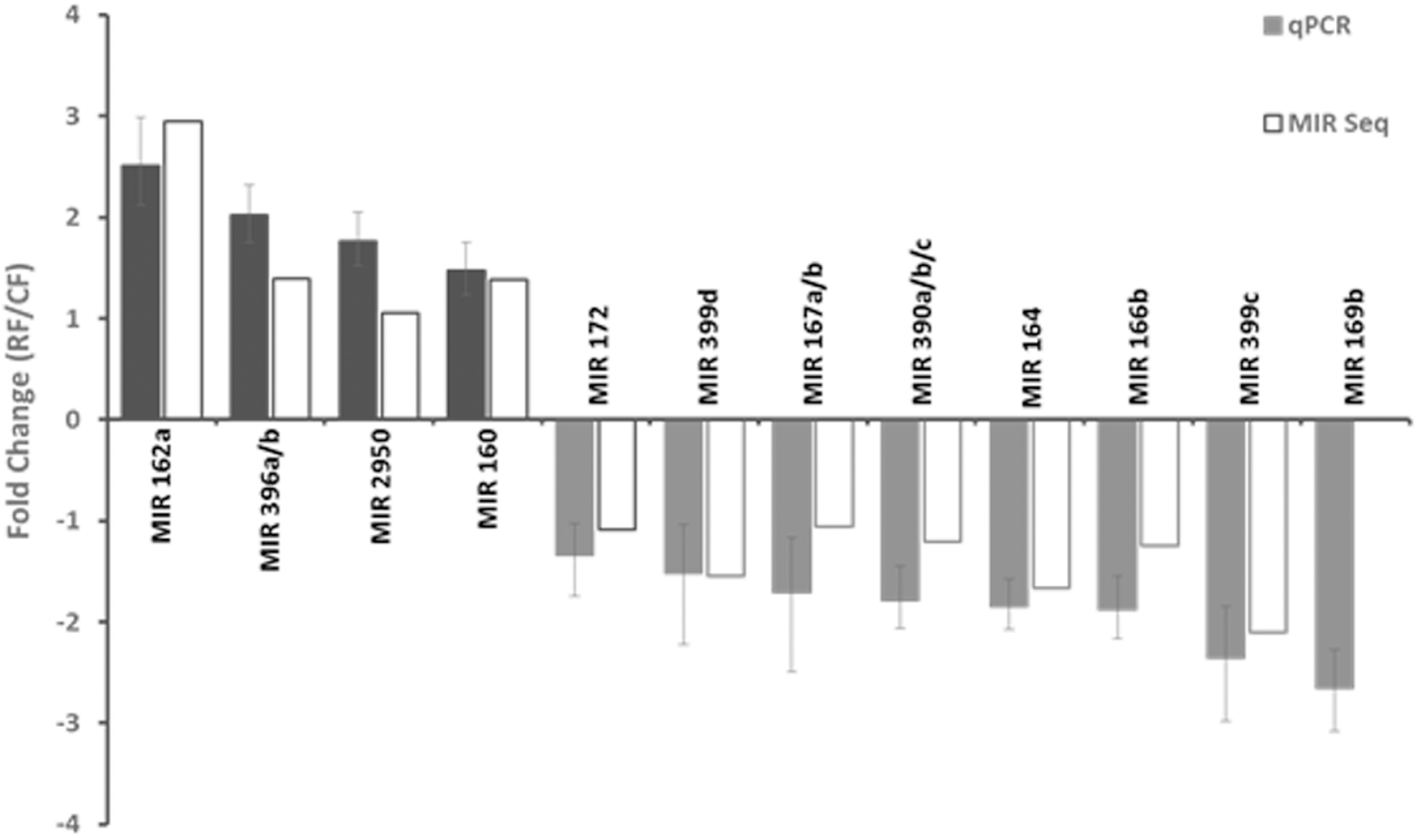
Validation of expression patterns of differentially-expressed-known miRNAs in 10 DPA-fiber of *PHYA1*-RNAi line by qRT-PCR. The miRNA expression was represented as relative fold change 2^-ΔΔCT^ (ΔΔC_T_ = ΔC_T RNAi_ - ΔC_T Coker 312_). The expression levels of miRNA were normalized by using U6 snRNA as a reference. Three biological replicates and three technical replicates were used for qRT-PCR analysis. The error bar represent the confidence limits. RF and CF denote fibers from RNAi and Coker 312 lines, respectively. MIR 169b was not detected in 10-DPA fiber by MIR Seq.

**Fig 3:**
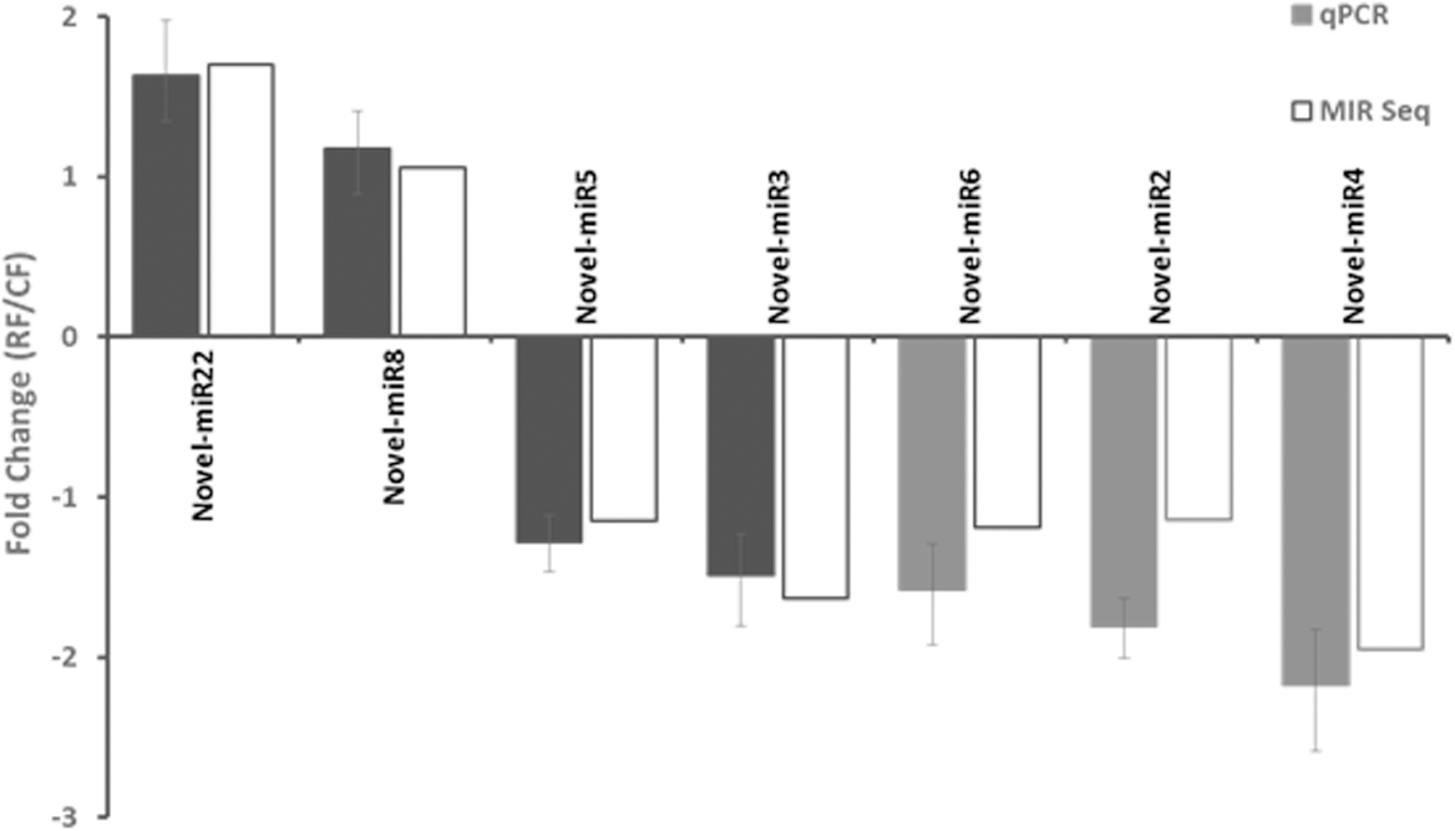
Validation of expression patterns of novel miRNAs in 10-DPA fiber of *PHYA1-* RNAi line by qRT-PCR. The miRNA expression was represented as relative fold change 2^-ΔΔCT^ (ΔΔC_T_ = ΔC_T RNAi_ - ΔC_T Coker 312_). The expression levels of miRNA were normalized by using U6 snRNA as a reference. Three biological replicates and three technical replicates were included for qRT-PCR assays. The error bar represent the confidence limits. RF and CF denote fibers from RNAi and Coker 312 lines, respectively.

The expression of twelve known miRNAs and seven novel miRNAs in 10-DPA fibers of *PHYA1* RNAi and Coker 312 lines was also determined by qRT-PCR (Figs 2 and 3, S1 Table). The abundances of these miRNAs were relatively high in majority of the fiber libraries (Fig 1). The results showed that these 19 miRNAs were successfully detected by qRT-PCR and showed consistency with the expression profiles analyzed by small RNA sequencing (Figs 2 and 3). Among the known miRNAs, miR162a, 396a/b, 2950 and 160 were up-regulated, and miR172, 399d, 167a/b, 390a/b/c, 164, 166b, 399c, and 169b were down-regulated (Fig 2). For the novel miRNAs, novel-miR22 and novel-miR8 were up-regulated, while novel-miR5, 3, 6, 2, and 4 were down-regulated (Fig 3). The expression level of these novel miRNAs (except for novel-miR4) in the *PHYA1* RNAi line was less than two-fold change when compared to Coker 312.

#### Inverse expression patterns between four pairs of miRNAs and their targets

**Fig 4:**
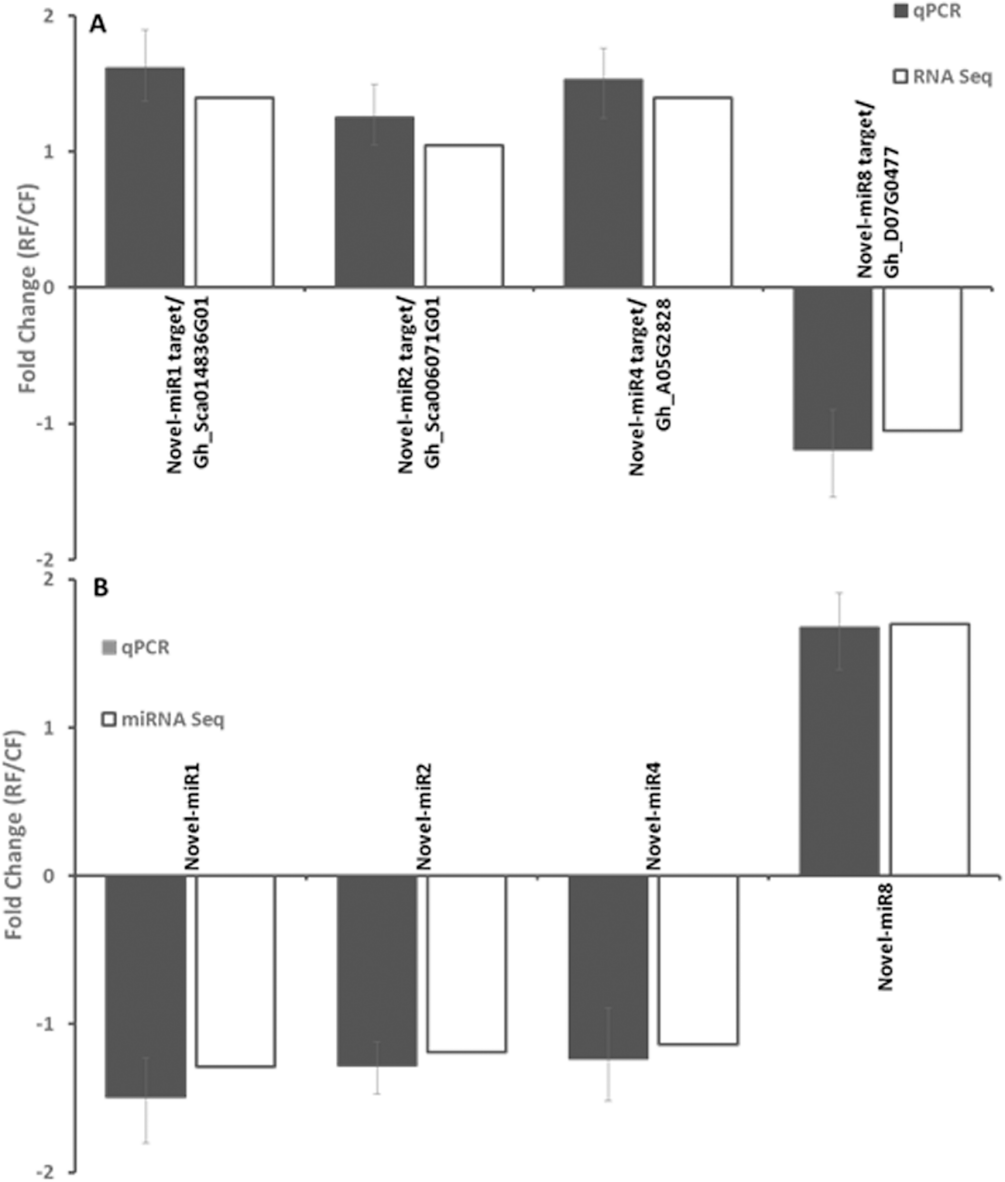
Inverse correlation between the expression of four novel miRNAs and their target genes. (A) RNA Sequencing and qRT-PCR analysis of predicted target genes of four novel miRNAs in 10 DPA fiber; (B) miRNA Sequencing and qRT-PCR analysis of four novel miRNAs in 10 DPA fiber. Gene expression was represented as fold change 2^-ΔΔCT^ (ΔΔC_T_ = ΔC_T RNAi_ - ΔC_T Coker 312_). The expression levels of the genes were normalized by *UBQ7* (Gen bank accession NO. DQ116441) and U6 snRNA as internal references for mRNAs and miRNAs, respectively. Three biological replicates and three technical replicates were included for qRT-PCR assays. The error bar represent the confidence limits.

Because miRNAs control the degradation of mRNA transcripts, the expression levels of miRNA and its target mRNA will be negatively correlated. To test this, the expression patterns of predicted targets of four novel miRNAs including novel-miR1, 2, 4 and 8 in 10 DPA fiber were analyzed by RNA sequencing and validated by qRT-PCR (Fig 4A). The results showed consistency between these two assays, and the relative expression levels of these miRNA targets were inversely correlated with the accumulation levels of corresponding miRNAs (Fig 4). For example, Gh_Sca142710G01 encoding an rRNA promoter binding protein was up-regulated in the *PHYA1* RNAi plant (Fig 4A) compared to Coker 312, and its corresponding miRNA novel-miR1 was down-regulated (Fig 4B). Novel-miR1 also targeted to cytochrome P450_TBP (The counts was too low to count in RNA Seq), which plays a crucial role in photosynthesis [49]. In addition to novel-miR1, eight of the novel miRNAs identified in upland cotton were predicted to target cytochrome P450 TBP (Table 3). This implied that the better quality fiber of the *PHYA1* RNAi line may be partially conferred through the miRNA regulation of cytochrome P450s, which are involved in a wide range of biosynthetic reactions, including fatty acid conjugation, hormones synthesis, and generating defensive compounds [50]. Thus, miRNA regulation on cytochrome P450 might play a positive role during fiber development.

## Discussion

Upland cotton (*Gossypium hirsutum* L.) produces the most commonly used textile fiber in the world. The improvement of cotton fiber quality using different genetics tools has long been a key interest for cotton breeders. Using RNAi technology, Abdurakhmonov et al. [2] had developed *PHYA1* RNAi cotton plants with improved fiber length, strength and fineness. Recently, the *GhMYB25* genes in allotetraploid cotton genome have been successfully targeted for mutagenesis using the CRISPR/Cas9 technology [51]. This finding indicates that the CRISPR/Cas9 system will be an excellent tool for functional analysis of cotton genes in A and D subgenomes and improvement of cotton fiber quality. In this study, a genome-wide miRNAome analysis was performed to identify differentially expressed miRNAs, which might reveal the molecular mechanisms of the fiber quality improvement by *PHYA1*RNAi in cotton. A total of 77 known miRNAs were identified in cotton fibers. Of these, 7 known miRNAs were differentially expressed (fold change ≥ 2) in the *PHYA1* RNAi plant. Using the published draft genome sequence of the allotetraploid cotton TM-1 line [30] as a reference, 35 novel cotton-specific miRNAs had been also identified and their target prediction was achieved.

### Identification of novel miRNAs in cotton

The utilization of allotetraploid TM-1 cotton genome as a reference is very useful for miRNA identification and target prediction. Without the complete genome sequence of *Gossypium hirsutum*, it is difficult to conduct miRNAome analysis through bioinformatics prediction. First of all, small RNA read libraries generated by previous studies might contain errors [52]. Secondly, it is hard to evaluate the possibility of MIR gene loci duplication in allotetraploid. Thirdly, the isolation/identification of miRNAs in cotton is lagging when compared to other plant species due to the complexity of cotton genome compensation. The major criterion for novel miRNA prediction is the hairpin structure formed by precursor miRNAs. Due to incomplete upstream and downstream sequences, it is difficult to predict the secondary structure of miRNAs [37]. Furthermore, the available transcript libraries in cotton research were mostly from *Gossypium raimondii*, and thus target prediction had generated extensive redundancy. In this study, the precursors were predicted and identified for all novel miRNAs by using the psRNATarget, and the redundancy of the targets was reduced by the second blasting with TM-1 as the reference, because many of the hits from distinct miRNAs turned out to be the same gene.

Using high-throughput small RNA sequencing, 89 conserved and 8 novel miRNAs have been recently identified in cotton [37]. Based on the annotation of whole genome sequence of the cultivated *Gossypium hirsutum* TM-1 upland cotton, 602 miRNAs [29] and 301 miRNAs [30] were identified, respectively, in the allotetraploid cotton by two research groups. As a result, further identification and functional validation of cotton miRNAs are still needed, particularly, for cotton specific miRNAs and fiber-development-related miRNAs. In this study, a total of 35 novel miRNAs were identified in the small RNA libraries of both RNAi and control genotypes. None of these novel miRNAs were differentially expressed in RNAi lines or was specific to RNAi genotype although there were subtle difference in expression levels. This is desired objective of any RNAi crop development that positively contributes to its biosafety concerns because if novel non-existed signature is generated by RNAi hairpins incretion into a genome, as a first step alert, RNAi product must be subjected for further risk assessment analyses [3].

Of the novel miRNAs, 16 miRNAs were in the D-subgenome, and another 15 miRNAs were in the A-subgenome. Most of miRNAs identified previously were located in the D-genome due to limited sequence information of upland cotton genomes [37]. An enlarged pool of sequenced genomes has increased the accuracy and coverage for bioinformatics prediction and analysis of miRNAs. The 24 nt miRNAs were the most abundant among the 35 novel miRNAs, which is consistent with previous studies [53, 54]. Unlike the 21 nt miRNAs associated with AGO1, the 24 nt miRNAs are loaded onto AGO4, which has a preference for sRNAs with 5´ terminal adenine [55, 56]. As expected, most of the 24 nt miRNAs (9) had a 5´ terminal adenine nucleotide, which is consistent with the previous study [54]. The predicted hairpin precursors had negative folding free energies from -45.7 to -230.1 kcal mol^-1^, which were in agreement with previous studies [55]. By base pairing to mRNAs, microRNAs mediate mRNA degradation or translational repression. Most of the novel miRNAs in this study inhibit target expression through mRNA degradation, and a few of them regulate the expression of the target genes through translational repression.

### Cotton fiber elongation-related miRNAs

In this study a total of 77 known miRNAs belonging to 61 miRNA families were identified in elongating fibers. Many of these conserved miRNAs were present at lower levels in RNAi cotton compared to Coker 312, including Gh-miR2950, 169b, 160, 399c and 399d. In contrast, Gh-miR7514, a cotton specific miRNA, was highly enriched in RNAi cotton. The target (ES793451) of Gh-miR2950, also a cotton specific miRNA, was predicted to encode a putative gibberellin 3 hydroxylase, which accumulated at higher levels in fibers [26]. Gibberellin 3 hydroxylase had been shown to control internode elongation in pea [57]. It was reported that Gh-miR2950 might affect fiber cell elongation via GA signaling [26]. The results in this study are consistent with this hypothesis, in that down-regulation of Gh-miR2950 promotes fiber elongation in the *PHYA1* RNAi cotton by increasing Gibberellin 3 hydroxylase activity and therefore increasing the levels of the biologically active gibberellin GA1 [58]. miRNA169, 160, 399 are highly conserved miRNAs in plants. It was reported that miR160 and miR169 were significantly expressed at low levels in fibers than in seedlings [59]. miRNA160 had been shown to target three auxin response factors ARF10, 16, and 17 [26]. It is well known that auxin plays an essential role in cotton fiber development [10, 11, 60]. The results in this study suggested that miR160 can promote fiber development via the auxin signaling transduction pathway through increased expression of ARF10, 16 and 17. miR399 was predicted to target a MYB transcription factor [54]. Many MYBs, such as MYB25 and 109, were reported to promote fiber elongation and their suppression caused shorter fibers. The ABA 8´ -hydroxylase catalyzed the first step of inactivation of ABA, and the active levels of ABA might increase by decreasing the expression of ABA 8´ -hydroxylase in the RNAi line. In this study, miR7514 was accumulated at higher levels in RNAi cotton, and it targeted the gene for Rho guanyl-nucleotide exchange factor 7 (RhoGEF7) [61]. The functional analysis of RhoGEF7 in plants has not been documented so far. However, it was reported that ABA could induce the degradation of another Rho guanyl-nucleotide exchange factor RhoGEF1 [62]. Taken together, the results in this study imply that miRNA mediated gene regulation may be involved in fiber improvement of the *PHYA1* RNAi cotton. Although the seven known miRNAs were differentially regulated in fiber cells and possibly involved in fiber development, some of them were found to play roles in biotic and abiotic stress responses. In wheat, miR160 was regulated under drought stress [63], miR164 was involved in salt stress response [64], and miR169b was differentially expressed in response to fungal infection [65]. In addition, miR396 in opium poppy was involved in regulation of secondary metabolite synthesis [66]. Another miRNA miRRNVL5 in *G*. *hirsutum* was found to play a role in regulation of cotton response to salt stress [67].

Cytochrome P450 TBP has the most abundant hits when the genome was screened with psRNATarget, and it was the target of 9 novel miRNAs identified in this study. Of these 9 miRNAs, 7 of them had lower levels in RNAi cotton compared to Coker 312. Cytochrome P450 is involved in the biosynthesis of plant hormones (such as ABA, GA, BR) and secondary metabolites (e.g. phenylpropanoids, alkaloids, terpreoids) [68]. Cytochrome P450 enzymes also play critical roles in response to different abiotic and biotic stresses. The cytochrome P450 involved pathways were abundantly present in RNAi cotton, and miR172, a stress related miRNA was reported to target Cytochrome P450 in cotton and corn [69, 70]. Taken together, although the expression of these novel miRNAs were not significantly changed in developing fibers, the observation of cytochrome P450 TBP as the target of 9 miRNAs suggests that miRNA mediated gene regulation on cytochrome P450 associated pathways might play a role in improving fiber quality in the *PHYA1* RNAi cotton.

### The possible functions of cytochrome P450 in cotton improvement

One possible explanation to the highest hits of cytochrome P450 TBP in miRNA target prediction is the high constitution of cytochrome P450 gene (CYP) family in plant genomes. It was reported that CYP genes occupied approximately 1% of the protein coding genes in six sequenced angiosperms, which include grape (*Vitis Vinifera*), papaya (*Carica papaya*), poplar (*Populus trichocarpa*), rice (*Oryza sativa*), Arabidopsis (*Arabidopsis thaliana*) and moss (*Physcomitrella patens*) and were involved in almost every aspects of the plant life [50]. Based on evolutionary analysis, the oldest CYPs are involved in biosynthesis of carotenoid and sterol. The middle branches of CYPs mediate the adaptation to environment, including abiotic stresses and biotic defenses. The newest CYPs are involved in the synthesis of structural components such as lignin, fatty acid, pigments, odorants, and signaling compounds [50]. Besides with improved fiber quality, *PHYA1* RNAi plants have vigorous root and vegetative growth, and early flowering due to increased photosynthesis [2, 3]. CYPs are light-driven oxidase enzymes in electron transfer chains [49]. Thus this study support the hypothesis that increased photosynthesis of RNAi cotton might be due to miRNA-mediated regulation on the expression of CYPs. In addition, CYPs are also involved in biosynthesis and catabolism of many plant hormones, such as ABA (CYP707), GA (CYP714) and BR (CYP724) [50]. ABA regulates osmotic stress tolerance [71], and GA plays a role in salinity adaptation [72]. As mentioned previously, CYPs also play crucial roles in environmental adaptation, which might explain the better tolerance of RNAi cotton to salinity, drought, and heat stresses.

## Conclusion

Eighteen RNA libraries constructed using small RNAs from 5-, 10-, and 15-DPA fibers of *PHYA1* RNAi and Coker 312 lines were sequenced using the Illumina HiSeq system. Sixty-one conserved miRNA families and thirty-five novel miRNAs were identified in the upland cotton lines. The targets of 6 conserved miRNAs, which expressed differentially in the RNAi line, were reported to participate in primary cell wall synthesis and phytohormone signaling pathways. The 35 novel miRNAs were identified in cotton for the first time, and their target genes were predicted. Nine novel miRNAs were identified to target cytochrome P450 TBP. Together, the results imply that miRNAs involved in fine-tune gene regulation might confer to the phenotype of the RNAi line with improved fiber quality.

## Supporting information

S1 Fig The small RNA length distribution in cotton fibers from both PHYA1 RNAi line (RF) and Coker 312 (CF). (TIF)

S1 Table Primers used in qRT-PCR. (DOCX)
