# Supplementary figures and images for: Genome-wide identification and characterization of microRNAs differentially expressed in fibers in a cotton phytochrome A1 RNAi line
Source: PLoS One. 2017 Jun 14;12(6):e0179381. doi: 10.1371/journal.pone.0179381 (PMC5470697; doi:10.1371/journal.pone.0179381)

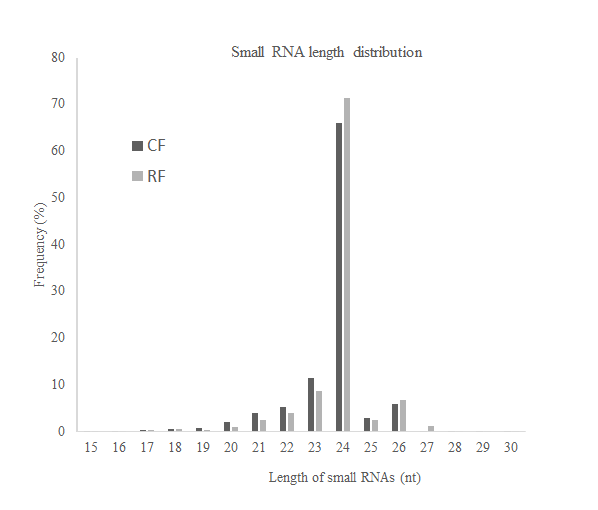

Supplement: S1 Fig — (TIF) [file pone.0179381.s001.tif]
