# Supplementary material for: Genome-wide identification and characterization of microRNAs differentially expressed in fibers in a cotton phytochrome A1 RNAi line
Source: PLoS One. 2017 Jun 14;12(6):e0179381. doi: 10.1371/journal.pone.0179381 (PMC5470697; doi:10.1371/journal.pone.0179381)
Supplement: S1 Table — (DOCX) [file pone.0179381.s002.docx]

**Table S1. Primers used in qRT-PCR**

| Quantitative RT-PCR Primers (5´ to 3´) | |
| --- | --- |
| MIR2950 | TGGTGTGCAGGGGGTGGAATA |
| MIR169b | CAGCCAAGGATGATTTGCCGG |
| MIR160 | TATGAGGAGCCATGCATGTAT |
| MIR399c | TGCCAAAGGAGAGTTGGCCTT |
| MIR399d | TGCCAAAGGAGATTTGCCCTG |
| Novel-mir-22 | TGTGTCAAATCGGCGGCTACATCT |
| Novel-miR-2 | CCGACTGTTTAATTAAAACAAAGT |
| Novel-miR-3 | GCGGCAAAATAGCTCGACGCCAGGAT |
| Novel-miR-4 | GGCTCAGCCGGAGGTAGGGTCCAG |
| Novel-miR-5 | CCGACCTTAGCTCAGTTGGTAGA |
| Novel-miR-6 | AAGAATTTGGGCTTTTGTGACTCG |
| Novel-miR-7 | GGCCAAGATCAATAGACAGGCGTG |
| Novel-miR-8 | TTCCACAGCTTTCTTGAACTT |
| MIR172 | AGAATCCTGATGATGCTGCAG |
| MIR390a/b/c | AAGCTCAGGAGGGATAGCGCC |
| MIR166b | TCGGACCAGGCTTCATTCCCC |
| MIR167a/b | TGAAGCTGCCAGCATGATCTA |
| MIR164 | TGGAGAAGCAGGGCACGTGCA |
| MIR396a/b | TTCCACAGCTTTCTTGAACTG |
| MIR162a | TCGATAAACCTCTGCATCCAG |
| Gh_Sca142710G01-F | CGACGTGCGGTGCTCTTC |
| Gh_Sca142710G01-R | CCACCCTGGAAACGGCTCA |
| Gh_Sca006071G01-F | GCCTCTCCGTCTCCCACTTC |
| Gh_Sca006071G01-R | AGACGGGCCAAAGGTCACAA |
| Gh_A05G2828-F | GCTGCTGGTGCACTGCTAGA |
| Gh_A05G2828-R | TGAGTCGACTGTTCGTTGCCT |
| GhD07G0477-F | GATGAAGCTGTCAAAGCCATTAG |
| GhD07G0477-R | CCAGTTGGTCCAGAGAAGATAAA |
